# Relationships between Cognitive Function and Odor Identification, Balance Capability, and Muscle Strength in Middle-Aged Persons with and without Type 2 Diabetes

**DOI:** 10.1155/2021/9961612

**Published:** 2021-10-07

**Authors:** Manabu Midorikawa, Hiroaki Suzuki, Yasuhiro Suzuki, Kazuyoshi Yamauchi, Hiroyuki Sato, Kiyotaka Nemoto, Yoko Sugano, Hitoshi Iwasaki, Motohiro Sekiya, Shigeru Yatoh, Naoya Yahagi, Yasushi Hada, Tetsuaki Arai, Hitoshi Shimano

**Affiliations:** ^1^Doctoral Program in Clinical Sciences, Graduate School of Comprehensive Human Sciences, University of Tsukuba, Tsukuba 305-8575, Japan; ^2^Department of Internal Medicine (Endocrinology and Metabolism), Faculty of Medicine, University of Tsukuba, Tsukuba 305-8575, Japan; ^3^Department of Rehabilitation Medicine, University of Tsukuba Hospital, Tsukuba 305-8596, Japan; ^4^Department of Laboratory Medicine, Faculty of Medicine, University of Tsukuba, Tsukuba 305-8575, Japan; ^5^Department of Psychiatry, Faculty of Medicine, University of Tsukuba, Tsukuba 305-8575, Japan; ^6^Department of Rehabilitation Medicine, Faculty of Medicine, University of Tsukuba, Tsukuba 305-8575, Japan; ^7^International Institute for Integrative Sleep Medicine (WPI-IIIS), University of Tsukuba, Tsukuba 305-8575, Japan; ^8^Life Science Center of Tsukuba Advanced Research Alliance (TARA), University of Tsukuba, Tsukuba 305-8575, Japan; ^9^Japan Agency for Medical Research and Development-Core Research for Evolutional Science and Technology (AMED-CREST), Chiyoda-ku 100-0004, Japan

## Abstract

**Aim:**

We investigated the relationship between cognitive function and olfactory and physical functions in middle-aged persons with and without type 2 diabetes (T2D) to examine the potential of olfactory and physical functions as biomarkers for early cognitive impairment.

**Methods:**

Enrolled were 70 T2D patients (age 40 to <65 y) and 81 age-matched control participants without diabetes. Cognitive function was assessed by the Montreal Cognitive Assessment (MoCA), Trail Making Test parts A and B (TMT-A/-B), Wisconsin Card Sorting Test (WCST), Quick Inventory of Depressive Symptomatology Self-Report (QIDS), and Starkstein Apathy Scale (SAS). Multiple linear regression analyses were performed.

**Results:**

Odor identification was an independent determinant shown in the results of the TMT-A in the entire participant group and was independently associated with the MoCA and TMT-B in the T2D group. Balance capability assessed with a stabilometer was independently associated with all cognitive function tests except for QISD and SAS in the entire participant group and the T2D group and was independently associated with TMT-A in the control group. Knee extension strength was independently associated with the SAS in the entire participant group and the T2D group.

**Conclusions:**

Odor identification, balance capability, and knee extension strength were potential markers for cognitive decline in middle-aged persons with T2D.

## 1. Introduction

The increase in dementia and its prevention and treatment are global concerns [1]. Although several drugs have been developed to improve cognitive decline in Alzheimer's disease (AD) [2], none has proven to be sufficiently effective [2]. It was reported that 16.5% of people with mild cognitive impairment (MCI), which is a precursor to dementia, develop AD annually [3], while about 20% recover to normal cognitive function [4]. Therefore, preventive interventions for persons with MCI or normal cognitive function who are at risk of developing dementia are considered to be important for dementia prevention.

Several modifiable risk factors for dementia have been reported, with diabetes being one of them [5]. In a systematic review of investigations of the development of dementia in persons with diabetes, the relative risk was 1.7–2.2 for AD and 2.2–2.8 for vascular dementia (VaD) [6]. Acute fluctuations of plasma glucose levels present a risk for dementia: elevated levels of 2-hour postload glucose in a 75 g oral glucose tolerance test were associated with the development of all-cause dementia, AD, and VaD in a population-based study [7], daily acute glucose fluctuations assessed with continuous glucose monitoring were reported to be independently associated with cognitive impairment in persons with type 2 diabetes (T2D) [8], and a cross-sectional study showed that visit-to-visit glucose variability was independently associated with the Mini-Mental State Examination score in elderly persons with T2D [9]. Although increased HbA1c levels and hypoglycemia have been thought to be risk factors for cognitive decline in diabetes, results of a systematic review were inconsistent [10]. In addition, cardiometabolic risk factors such as hypertension, dyslipidemia, and obesity, which are often associated with diabetes, are risk factors for cognitive decline in the general population [11].

Numerous studies have reported on associations between olfactory or physical dysfunction and cognitive decline. Olfactory dysfunction, especially odor identification, was observed in persons with MCI and AD [12, 13] and was more prominent in AD compared with MCI [13, 14]. The results of an odor identification score were significantly associated with AD pathology based on counts of cortical plaques and tangles or density of tau-positive neurofibrillary tangles in the entorhinal cortex and hippocampus [15]. With regard to physical function, both gait speed and balance capability were significantly associated with cognitive function in elderly people [16]. A systematic review showed that changes in grip strength were associated with changes in cognitive function, although this association was inconsistent [17]. Impaired lower extremity function assessed by walking speed and the number of steps taken within a designated time, standing up from a chair, and balancing was significantly associated with the development of AD in persons with MCI, whereas there was no significant association between dexterity and the development of AD [18]. Furthermore, olfactory function was shown to be associated with motor function in elderly persons [19].

Relationships among cognitive function, olfactory function, and physical function have also been reported in those with diabetes. A systematic review showed that persons with diabetes had significant olfactory impairment compared with control participants [20]. Furthermore, the olfactory identification score was an independent determinant of cognitive impairment assessed with the Mini-Mental State Examination in elderly persons with T2D [21]. Both timed walk and grip strength were independent determinants of performance in all cognitive domains except for episodic memory in elderly persons with T2D [22].

A prospective cohort study showed that a younger onset of T2D was significantly associated with an increased risk of dementia: at the age of 70 years, the hazard ratio of dementia for every 5-year increment of earlier onset of T2D was 1.24 [23]. Therefore, interventions that take cognitive decline into account are important in middle-aged persons with T2D and early detection of persons with T2D who are at higher risk of cognitive decline may be useful in devising strategies to prevent or delay the subsequent onset of dementia. Although the identification of noninvasive biomarkers, such as olfactory function and physical function, is important for the early prevention of cognitive decline in persons with T2D, the relationship between cognition and olfactory function and physical function in middle-aged persons with T2D has not been reported. Nor has such relationships been reported in middle-aged persons without diabetes. The clinical questions in the current study were (i) whether olfactory function, balance capability, and muscle strength were associated with cognitive function even in the middle-aged adults, (ii) whether those associations were different between persons with diabetes and those without diabetes, and (iii) which physical function was important for each cognitive domain.

It must be recognized that testing methods used in studies of the elderly of balance capability using one-leg standing time (OLST) or the Timed Up and Go Test (TUG) and muscle strength testing using hand-held dynamometry may be so easy for middle-aged persons that subtle differences among groups or participants cannot be detected through these means. We aimed at investigating the relationships between cognitive function and olfactory function and physical function in middle-aged persons with T2D and nondiabetic controls to clarify the potential of these factors as biomarkers for early detection of cognitive impairment. In addition, we examined whether precise balance and muscle strength assessments using a stabilometer and a torque machine could be useful in predicting cognitive dysfunction in middle-aged adults with and without T2D.

## 2. Methods

### 2.1. Participants

From November 2017 to August 2020, we enrolled 70 persons with T2D and 81 nondiabetic control participants who were 40 to <65 years old and had no clinically apparent cognitive impairment. The diabetic persons were recruited from those who had visited outpatient clinics or were admitted to hospital for treatment of diabetes. Nondiabetic control participants were recruited using our department's website, bulletin boards at our university, and a local community magazine. Exclusion criteria included (i) history of central nervous system disorders, head injury, psychiatric disorders, or olfactory disorders; (ii) rhinitis; (iii) use of drugs affecting neuropsychological function; and (iv) being unable to walk independently without assistive devices. No one was excluded based on results of cognitive function tests. Written informed consent was obtained from participants prior to study enrollment. This study was conducted in accordance with the principles of the Declaration of Helsinki and approved by the Clinical Research Ethics Committee of University of Tsukuba Hospital (H29-129).

### 2.2. Clinical Evaluation

Participants were surveyed for age, sex, smoking habits, years of education, systolic blood pressure, diastolic blood pressure, physical activity, and medication status. In addition, in persons with T2D, information was obtained on the duration of diabetes and diabetic complications. All participants were asked about the frequency, amount, and type of alcohol that they currently consumed. The amount of physical activity was calculated using the International Standardized Physical Activity Questionnaire [24]. Body composition was evaluated by bioelectrical impedance analysis (InBody 720, BioSpace, Tokyo). Body mass index (BMI) and skeletal muscle mass index were calculated by dividing the body weight (kg) by the square of the height (m^2^) and dividing the limb skeletal muscle mass (kg) by the square of the height (m^2^), respectively.

### 2.3. Assessment of Cognitive Function

Global cognitive function was assessed using the Japanese version of the Montreal Cognitive Assessment (MoCA) [25]. Originally, participants with <12 years of education would receive an additional 1 point on the MoCA score to assess cognitive function; however, we used raw data because age-adjusted scores were reported to be less sensitive in detecting cognitive decline [26]. Trail Making Test parts A and B (TMT-A/-B) were used for assessing processing speed and executive function, respectively [27]. The personal computer version of the Wisconsin Card Sorting Test Keio version (KWCST) [28], which is a simplified version of the Wisconsin Card Sorting Test (WCST), was used to assess frontal lobe functions such as cognitive flexibility and abstract concepts. The number of categories achieved (CA) and perseverative errors of the Nelson type (PEN) were extracted. Depressive symptoms were assessed using the Japanese version of the Quick Inventory of Depressive Symptomatology Self-Report (QIDS) [29]. The Japanese version of the Starkstein Apathy Scale (SAS) was used to evaluate motivation [30].

### 2.4. Assessment of Olfactory Function

Olfactory function was evaluated using a card-type odor identification test developed for Japanese people (Open Essence^Ⓡ^, Fuji Film Wako Pure Chemicals, Tokyo, Japan). Open Essence consists of 12 kinds of odorants, which are perfume, rose, condensed milk, mandarin orange, curry, roasted garlic, sweat-smelling clothes, cooking gas, menthol, India ink, wood, and Japanese cypress [31]. Total scores ranged from 0 to 12 points.

### 2.5. Assessment of Physical Function

Balance capability was evaluated with the OLST, TUG, and index of postural stability (IPS). OLST was measured with both eyes open and also with both eyes closed. The test was performed twice with the examinee standing on each leg, and the longest time was considered to be the representative value. The maximum measurement time was 120 seconds with the eyes open and 60 seconds with the eyes closed. TUG was performed as follows: the participant stood up from a chair without elbow rests and walked 3 meters forward and back as quickly as possible [32]. IPS was measured using a stabilometer (GP-6000, Anima, Tokyo, Japan) as described elsewhere [33]. First, participants stood in a resting position with the inside of the foot at a distance of 10 cm on the stabilometer to measure instantaneous fluctuations in the center of pressure (COP) at a sampling frequency of 20 Hz. Then, participants were instructed to incline the body to the front, rear, right, and left keeping the body straight and without moving the feet. The instantaneous fluctuations in COP were measured at each position. IPS was calculated as “log [(area of stability limit + area of postural sway)/area of postural sway].” The area of the stability limit was calculated as the “front and rear center movement distance between anterior and posterior positions × the distance between right and left positions.” The area of postural sway was calculated as “average measurement value in 10 seconds under anterior, posterior, right, left, and center positions.” The area of postural sway was calculated as the mean sway area of the 5 positions.

Muscle strength was evaluated according to grip strength, knee extension muscle strength, and knee extension muscle endurance. The grip strength of the dominant hand was measured using a Smedley analog grip strength meter (Toei Light, Saitama, Japan). Knee extension muscle strength and knee extension muscle endurance were evaluated on the dominant foot side using a torque machine (Biodex System 3, Sakai Medical, Tokyo, Japan). For knee extension muscle strength, the participant performed three consecutive knee extension operations with maximum effort in isokinetic muscle strength measurement (60°/s) and the maximum torque value adjusted by body weight was used as a representative measurement value. Knee extension muscle endurance was measured by the total work from 20 continuous knee extensions with maximum effort by isokinetic muscle strength measurements (300°/s).

### 2.6. Laboratory Analysis

Blood samples were collected in the morning after an overnight fast. Plasma glucose and total cholesterol, low-density lipoprotein cholesterol, high-density lipoprotein cholesterol, and triglycerides were determined using an automated analyzer (Hitachi High-Technologies, Tokyo, Japan). HbA1c was measured by high-performance liquid chromatography (TOSOH, Tokyo, Japan). The apolipoprotein E (ApoE) genotype was analyzed by a polymerase chain reaction-restriction fragment length polymorphism method [34].

### 2.7. Statistical Analysis

Based on distribution, continuous variables were expressed as mean ± standard deviation or median (interquartile range) and compared using the unpaired *t*-test or the Mann–Whitney *U*-test for two-group comparisons. Categorical variables are expressed as numerals and percentages and were compared with Fisher's exact test. Spearman's rank correlation coefficient was used to examine bivariate associations between tests of cognitive function and olfactory or physical functions. A multiple linear regression analysis was performed to examine whether olfactory function, or physical functions were independent determinants of cognitive functions. We adopted IPS and knee extension strength as explanatory variables representative of balance capability and muscle strength based on results of the correlation analysis in the overall, T2D, and control groups. TMT-A, TMT-B, PEN in KWCST, and QIDS were log transformed, and MoCA was cubed due to their nonnormal distribution. Because the scores of PEN and QIDS contain 0, we added 1 to the scores of PEN and QIDS before log transformation. The ApoE genotype was categorized as either an *ε*4 carrier or noncarrier. Statistical analyses were performed using SPSS Statistics 26 (Chicago, IL, USA). Statistical significance was considered at a *P* value of <0.05.

## 3. Results

Table 1 shows the clinical characteristics of the study participants. The T2D group had significantly higher proportions of smokers, hypertension, and dyslipidemia; significantly lower proportions of females and ApoE *ε*4 allele carriers; and significantly shorter education periods compared with the control group. Age, total physical activity, and alcohol consumption were not significantly different between the two groups. As to body composition, BMI, body fat percentage, and SMI were significantly higher and skeletal muscle percentage was significantly lower in the T2D group than in the control group.  Table 2 shows the results of cognitive function tests, odor identification test, and physical function tests. Results of cognitive function testing and tests of knee extension strength, endurance, and balance capability in the T2D group were significantly worse than those in the control group. Grip strength did not differ significantly between the two groups.

The results of the correlation analysis between the cognitive function tests and odor identification test, balance capability, and muscle strength were as follows: (i) the odor identification score was significantly correlated with MoCA, TMT-A, and QIDS (Table 3) and with MoCA and TMT-B in the T2D group (Table 4). None of the cognitive subdomains was correlated with the odor identification score in the control group (Table 5), (ii) balance capability-related variables were significantly correlated with all of the cognitive subdomains in the entire cohort (Table 3), with MoCA, TMT-A and -B, CA, and PEN in WCST in the T2D group (Table 4) and with a few cognitive subdomains in the control group (Table 5), and (iii) muscle strength-related variables had significant correlations with all of the cognitive subdomains except for MoCA in the entire cohort (Table 3), with TMT-A and -B, and CA, and PEN in WCST in the T2D group (Table 4) and with TMT-B, PEN in WCST, and SAS in the control group (Table 5).

In the entire participant group, the results of multiple linear regression analyses adjusted by age, sex, education (≤12 years or >12 years), presence of diabetes, hypertension, ApoE *ε*4 carrier, and BMI were as follows (Table 6): (i) results of the odor identification test were significantly associated with TMT-A and tended to be associated with MoCA and QIDS, (ii) IPS was independently associated with all cognitive function tests except for QIDS and SAS, and (iii) knee extension strength was significantly associated with TMT-B and SAS.  Table 7 shows the results of multiple linear regression analysis adjusted for age, sex, education, and ApoE *ε*4 carrier status in the control group and the T2D group. In the control group, IPS was independently associated with TMT-A and tended to be associated with MoCA and SAS. However, the odor identification test and tests of knee extension strength did not show a statistically significant correlation with any of the cognitive function test results in the control group. In contrast, in the T2D group, results of the odor identification test had independent associations with MoCA and TMT-B, IPS was independently associated with MoCA, TMT-A and -B, and CA and PEN in WCST, and knee extension strength had an independent association with SAS.

## 4. Discussion

The aim of this study was at examining the associations between cognitive function and olfactory and physical functioning in middle-aged persons with and without T2D and at determining whether olfactory and physical functions could be biomarkers for early detection of cognitive impairment. It is also aimed at examining whether precise balance and muscle strength assessments using a stabilometer and a torque machine could be useful in predicting cognitive function in middle-aged adults. There were four major findings in the current study. First, results of all measurements of cognitive function, odor identification, balance capability-related variables, and muscle strength-related variables in middle-aged persons with T2D were significantly worse than those in the age-matched controls. Second, balance capability measured by IPS was independently associated with all of the measured cognitive function tests except for QIDS and SAS in the entire cohort and the T2D group. Third, knee extension strength was significantly associated with SAS in the entire cohort and in the T2D group. Fourth, results of the odor identification test were independently associated with TMT-A in the entire cohort and with MoCA and TMT-B in the T2D group.

### 4.1. Cognitive Function and Physical Function in Middle-Aged Persons with T2D

Previous reports showed decreased memory, attention, executive function, and information processing ability and increased prevalence of depression and apathy even in middle-aged patients with T2D [35–37]. In addition, decreased olfactory function, balance capability, and lower limb muscle strength have been shown in those with T2D [38–40]. Although sex and years of education differed significantly between the T2D group and the control group, those findings are consistent with our results; global cognition (MoCA), processing speed (TMT-A), executive function (TMT-B), cognitive flexibility (CA in WCST), perseveration (PEN in WCST), depressive symptoms (QIDS), motivation (SAS), odor identification, balance capability, and muscle strength in the T2D group were significantly worse than those in the control group. Those findings were also consistent even when the two participant groups were matched for age, sex, and years of education (Table 8). Although the proportion of ApoE *ε* 4 carriers was significantly higher in the control group than in the T2D group, both cognitive function and physical performance were significantly reduced in persons with T2D compared to nondiabetic individuals, suggesting that interventions to prevent cognitive and physical decline may be needed even in middle-aged persons with T2D.

### 4.2. Relationship between Cognitive Function and Physical Function

In the entire cohort of the current study, balance capability assessed by IPS was significantly associated with global cognition, processing speed, executive function, cognitive flexibility, and perseveration after adjustment for confounding factors. It was shown that the score of the MoCA was significantly associated with OLST with eyes open in community dwelling elderly persons [41]. Also, OLST with eyes open was reported to be significantly associated with memory, processing speed, and executive function in individuals aged 45 to 85 years (mean age 62.9 years) [42]. Tangen et al. reported that balance ability assessed with the Balance Evaluation Systems Test deteriorated in conjunction with increasing severity of cognitive impairment, especially in executive function, in individuals with subjective cognitive impairment, MCI, and AD [43]. Using a stabilometer, it was shown that the length of anteroposterior postural sway [44] or mean velocity of postural sway at the COP [45] was increased with the severity of cognitive impairment in elderly persons. Moreover, the sway path length was significantly correlated with supratentorial cerebrospinal fluid volume, white matter hyperintensities volume, and the Dementia Rating Scale [46].

There are few reports on the association between balance capability and cognitive function in T2D. Smith and colleagues showed that in elderly persons with T2D, executive function and reduced postural stability under dual task conditions were worse compared with those in age-matched controls [47]. In the current study, the IPS was the best balancing test in comparison with other balancing tests in terms of showing the broadest association with the cognitive domains measured (data not shown). The IPS is characterized by high correlation with the Berg balance scale, high reproducibility, and not having a ceiling effect [33]. In addition, the degree of difficulty in the IPS may have been suitable compared with other balance tests in middle-aged individuals.

There have been several reports on the association between cognitive function and lower limb muscle function [41, 48–50]. A study of community-dwelling older adults with no apparent cognitive impairment showed that handgrip strength, leg strength, sit-to-stand ratio, gait speed, and one-leg standing time were all significantly associated with MoCA scores in multiple regression analysis [41]. It was also reported that knee extension strength was significantly associated with executive function as assessed with the digit symbol substitution test after adjusting for confounding factors in individuals aged 60 years or older [48]. Steves and colleagues reported that leg power was significantly associated with both a 10-year cognitive decline and subsequent total grey matter volume in female twins [49]. A prospective study showed that physical activity in persons with apathy was significantly lower than that in persons without apathy and that apathy was associated with a decline in physical performance in elderly persons [50]. In the current study, knee extension strength was significantly associated with executive function and motivation after adjusting for confounding factors and was a better determinant of executive function and motivation than knee extension endurance (data not shown). No studies have examined the relationship between cognitive function and both knee extension strength and knee extension endurance. Further research on this topic is needed. The significant association between SAS and knee extension strength in the current study may indicate that low physical activity due to apathy causes low muscle strength.

### 4.3. Relationship between Cognitive Function and Olfactory Function

A study using positron emission tomography showed that the hippocampus, orbitofrontal cortex, amygdala, parietal cortex, insula, cerebellum, right temporal cortex, and parietal cortex were activated in odor discrimination and memory [51]. In persons with T2D, activation of the left hippocampus and left parahippocampus in response to odor stimuli was significantly reduced as was functional brain connectivity in the right inferior and middle orbitofrontal cortex compared with healthy individuals [52]. Although few reports have examined the relationship between olfactory function and cognitive function in middle-aged individuals, olfactory function has been suggested to be significantly associated with several cognitive domains [52, 53]. Zhang et al. reported that olfactory behavior scores were significantly correlated with MoCA, word fluency, and executive function in persons with T2D but only significantly correlated with episodic memory in the control group [52]. Schubert et al. showed that olfactory impairment was significantly associated with poor performance on the TMT-A, TMT-B, and Grooved Pegboard in middle-aged adults [53]. The current study showed that odor identification was independently associated with TMT-A and had a tendency to be associated with MoCA and QIDS after adjustment for confounding factors in the entire cohort. Furthermore, the odor identification score was independently associated with MoCA and TMT-B in the T2D group but not in the control group. The results of the current study were consistent with the report of Zhang et al. [52]. Because the sample size of the control group in this study was smaller and performance on the TMT-A and TMT-B was better compared with a previous report [53], it is possible that there was no significant association between odor identification scores and results of the TMT-A and TMT-B. Impairment of olfactory identification could be a surrogate marker of decreased frontal lobe function, which is responsible for information processing and executive functions [54].

### 4.4. Clinical Implications

The current study showed that several cognitive domains were significantly associated with odor identification, balance capability, and lower limb muscle strength. These physical tests are noninvasive and low cost. Testing of these physical abilities may identify groups at high risk for cognitive decline and allow for early detection and interventions. In particular, in multiple linear regression analyses, IPS was the strongest risk factor among a wide range of cognitive function domains compared to other risk factors. Therefore, IPS may be useful in predicting cognitive decline in those with and without T2D.

## 5. Limitations

This study has several limitations. First, the presence of organic brain diseases such as asymptomatic cerebral infarction could not be ruled out because the participants did not undergo magnetic resonance imaging. Second, the T2D group had a significantly shorter duration of education and included a significantly lower proportion of women and ApoE *ε*4 carriers compared with the control group. Although we conducted multiple linear regression analyses, we cannot rule out the possibility that the effects of those imbalances were not fully corrected for. Third, this study did not consider the effects of antidiabetic drugs on cognitive function. Several antidiabetic drugs have been reported to affect cognitive function and olfactory function in animal studies and in human trials [55–59]. The inclusion of each antidiabetic drug as an explanatory variable instead of sex had no effect on the association between olfactory and physical function and cognitive function, except that metformin abolished the significance of knee extension strength in SAS (data not shown). In addition, the small sample size did not allow us to include those drugs in the model simultaneously without eliminating other possible risk factors for dementia. Finally, since this is a cross-sectional study with a small number of cases, it is not clear whether odor identification, balance capability, or lower muscle strength can predict future cognitive decline. It is necessary to conduct a longitudinal study with a larger study population.

## 6. Conclusions

In conclusion, middle-aged persons with T2D had lower cognitive function, olfactory function, balance capability, and lower extremity muscle strength than the nondiabetic controls. Odor identification, balance capability assessed with a stabilometer, and knee extension strength assessed by a torque machine were independent risk factors for cognitive decline in the middle-aged study participants with T2D. These findings could be useful for early detection of cognitive decline.

## Figures and Tables

**Table 1:** Clinical characteristics of study participants.

	All (n = 151)	Controls (n = 81)	Type 2 diabetes (n = 70)	*P*
Age (years)	53 ± 7	52 ± 6	53 ± 7	0.323
Female, n (%)	75 (50)	47 (58)	28 (40)	0.020
Education (y)	14 (12 – 16)	15 (14 – 16)	12 (12 – 14)	<0.001
ApoE *ε*4 allele carrier, n (%)	29 (19)	22 (27)	7 (10)	0.006
Current Smoking, n (%)	25 (17)	5 (6)	20 (29)	<0.001
Alcohol consumption (g/d)	6 (0 – 20)	7 (0 – 20)	0 (0 – 20)	0.092
Total physical activity (MET-min/w)	1724 (656 – 4064)	2010 (735 – 4299)	1253 (401 – 3803)	0.098
Body mass index (kg/m^2^)	24.6 (21.9 – 28.4)	22.1 (21.1 – 24.5)	28.2 (25.4 – 30.1)	<0.001
Body fat percentage (%)	29.9 ± 8.8	26.6 ± 7.7	33.8 ± 8.4	<0.001
Skeletal muscle percentage (%)	38.4 ± 5.3	40.3 ± 4.7	36.3 ± 5.1	<0.001
Skeletal muscle mass index (kg/m^2^)	7.2 (6.4 – 8.0)	6.9 (6.1 – 7.6)	7.6 (6.7 – 8.5)	<0.001
Fasting plasma glucose (mmol/L)	5.8 (5.1 – 8.7)	5.1 (4.9 – 5.4)	8.9 (7.7 – 10.3)	<0.001
HbA1c (%)	6.0 (5.6 – 8.5)	5.6 (5.4 – 5.8)	8.9 (7.6 – 11.1)	<0.001
HbA1c (mmol/mol)	42 (38 – 69)	38 (36 – 40)	74 (60 – 98)	<0.001
Total cholesterol (mmol/L)	5.2 ± 0.9	5.5 ± 0.7	4.9 ± 1.0	<0.001
HDL-C (mmol/L)	1.6 ± 0.5	1.9 ± 0.4	1.2 ± 0.2	<0.001
LDL-C (mmol/L)	3.0 ± 0.7	3.1 ± 0.6	3.0 ± 0.8	0.618
Triglycerides (mmol/L)	1.1 (0.8 – 1.7)	0.8 (0.7 – 1.1)	1.7 (1.2 – 2.3)	<0.001
Systolic blood pressure (mmHg)	127 (116 – 138)	125 (115 – 135)	129 (118 – 142)	0.084
Diastolic blood pressure (mmHg)	80 ± 12	81 ± 12	79 ± 12	0.450
Hypertension, n (%)	35 (23)	7 (9)	28 (40)	<0.001
Dyslipidemia, n (%)	48 (32)	3 (4)	45 (64)	<0.001
Duration of diabetes (y)			7.0 (2.0 – 11.0)	
Diabetic complications				
Retinopathy, n (%)			18 (26)	
Nephropathy, n (%)			23 (33)	
Peripheral neuropathy, n (%)			26 (37)	
Cardiovascular disease, n (%)			8 (11)	
Antidiabetic drugs				
Metformin, n (%)			43 (61)	
Sulfonylureas, n (%)			15 (21)	
Glinides, n (%)			1 (1)	
Thiazolidinediones, n (%)			7 (10)	
Sodium-glucose cotransporter 2 inhibitors, n (%)			22 (31)	
Dipeptidyl peptidase-4 inhibitors, n (%)			33 (47)	
Glucagon like peptide-1receptor agonists, n (%)			6 (9)	
Alpha-glucosidase inhibitors, n (%)			3 (4)	
Insulin, n (%)			16 (23)	

Data are mean ± SD or median (interquartile range). ApoE: apolipoprotein E; HDL-C: high-density lipoprotein-cholesterol; LDL-C: low-density lipoprotein-cholesterol.

**Table 2:** Comparison of cognitive function tests, odor identification test, and physical function tests.

	All(n = 151)	Controls (n = 81)	Type 2 diabetes (n = 70)	*P*
Cognitive function				
MoCA (points)	26 (24 – 28)	27 (26 – 28)	25 (22 – 27)	<0.001
TMT-A (s)	29.4 (23.0 – 34.6)	25.9 (21.4 – 31.9)	31.7 (25.8 – 39.0)	0.001
TMT-B (s)	66.1 (55.1 – 90.4)	63.3 (52.6 – 80.7)	79.3 (60.0 – 109.0)	<0.001
WCST				
CA (points)	4 (2 – 5)	4 (3 – 5)	3 (1 – 5)	0.005
PEN (points)	5 (2 – 12)	3 (2 – 7)	9 (4 – 15)	<0.001
QIDS (points)	4 (2 – 7)	3 (2 – 6)	5 (3 – 9)	<0.001
SAS (points)	11 ± 6	9 ± 5	13 ± 6	<0.001
Olfactory function				
Open Essence (points)	9 (8 – 10)	10 (8 – 11)	9 (8 – 10)	<0.001
Physical function				
OLST (eyes open) (s)	120 (86 – 120)	120 (120 – 120)	120 (37 – 120)	<0.001
OLST (eyes closed) (s)	13 (6 – 28)	21 (10 – 39)	7 (4 – 14)	<0.001
TUG (s)	5.5 ± 1.0	5.0 ± 0.7	6.1 ± 0.8	<0.001
IPS	1.80 (1.63 – 1.96)	1.92 (1.81 – 2.02)	1.64 (1.47 – 1.76)	<0.001
Grip strength (kg)	31 (25 – 41)	31 (27 – 41)	29 (24 – 41)	0.202
Knee extension strength (Nm/kg)	190 ± 49	211 ± 44	166 ± 43	<0.001
Knee extension endurance (J)	1053 (806 – 1353)	1081 (896 – 1415)	937 (715 – 1258)	0.009

Data are mean ± SD or median (interquartile range). CA: categories achieved; IPS: index of postural stability; MoCA: Montreal Cognitive Assessment; OLST: one-leg standing time; PEN: perseverative errors of Nelson; QIDS: Quick Inventory of Depressive Symptomatology Self-Report; SAS: Starkstein Apathy Scale; TMT-A: Trail Making Test part A; TMT-B: Trail Making Test part B; TUG: Timed Up and Go Test; WCST: Wisconsin Card Sorting Test.

**Table 3:** Correlation between cognitive function tests and olfactory identification test, balance capability tests, and muscle strength tests in the entire participants.

	MoCA	TMT-A	TMT-B	WCST (CA)	WCST (PEN)	QIDS	SAS
Open essence	**0.216∗∗**	−**0.191∗**	−0.157	0.128	−0.145	−**0.210∗**	−0.069
OLST (eyes open)	**0.288∗∗∗**	−**0.377∗∗∗**	−**0.286∗∗∗**	**0.208∗**	−**0.249∗∗**	−0.141	−**0.268∗∗∗**
OLST (eyes closed)	**0.294∗∗∗**	−**0.326∗∗∗**	−**0.227∗∗**	**0.208∗**	−**0.223∗∗**	−**0.252∗∗**	−**0.266∗∗∗**
TUG	−**0.275∗∗∗**	**0.262∗∗**	**0.258∗∗**	−**0.181∗**	**0.305∗∗∗**	**0.260∗∗**	**0.296∗∗∗**
IPS	**0.442∗∗∗**	−**0.308∗∗∗**	−**0.385∗∗∗**	**0.263∗∗**	−**0.338∗∗∗**	−**0.249∗∗**	−**0.238∗∗**
Grip strength	−0.051	−**0.160∗**	−0.142	**0.233∗∗**	−**0.203∗**	−0.063	−**0.212∗∗**
Knee extension strength	0.154	−**0.288∗∗∗**	−**0.301∗∗∗**	**0.242∗∗**	−**0.347∗∗∗**	−**0.170∗**	−**0.325∗∗∗**
Knee extension endurance	0.108	−**0.253∗∗**	−**0.296∗∗∗**	**0.223∗∗**	−**0.308∗∗∗**	−0.058	−**0.223∗∗**

^∗^ *P* < 0.05, ^∗∗^*P* < 0.01, and ^∗∗∗^*P* < 0.001. CA: categories achieved; IPS: index of postural stability; MoCA: Montreal Cognitive Assessment; OLST: one-leg standing time; PEN: perseverative errors of Nelson; QIDS: Quick Inventory of Depressive Symptomatology Self-Report; SAS: Starkstein Apathy Scale; TMT-A: Trail Making Test part A; TMT-B: Trail Making Test part B; TUG: Timed Up and Go Test; WCST: Wisconsin Card Sorting Test.

**Table 4:** Correlation between cognitive function test and olfactory identification, balance capability, and muscle strength in the type 2 diabetes group.

	MoCA	TMT-A	TMT-B	WCST (CA)	WCST (PEN)	QIDS	SAS
Open Essence	**0.264∗**	−0.140	**−0.244∗**	0.014	−0.028	−0.129	−0.078
OLST (eyes open)	0.149	**−0.378∗∗**	**−0.239∗**	0.202	−0.227	0.121	−0.091
OLST (eyes closed)	**0.264∗**	**−0.372∗∗**	**−0.315∗∗**	**0.291∗**	−0.180	0.032	−0.123
TUG	−0.124	0.175	**0.264∗**	−0.124	**0.300∗**	−0.090	0.152
IPS	**0.415∗∗∗**	−0.187	**−0.436∗∗∗**	**0.374∗∗**	**−0.352∗∗**	−0.036	0.044
Grip strength	−0.104	−0.076	−0.011	**0.334∗∗**	**−0.308∗∗**	0.060	−0.147
Knee extension strength	−0.016	−0.164	−0.132	0.179	**−0.279∗**	0.029	−0.149
Knee extension endurance	0.055	**−0.239∗**	**−0.260 ∗**	0.189	**−0.272∗**	0.078	−0.123

^∗^ *P* < 0.05, ^∗∗^*P* < 0.01, and ^∗∗∗^*P* < 0.001. CA: categories achieved; IPS: index of postural stability; MoCA: Montreal Cognitive Assessment; OLST: one-leg standing time; PEN: perseverative errors of Nelson; QIDS: Quick Inventory of Depressive Symptomatology Self-Report; SAS: Starkstein Apathy Scale; TMT-A: Trail Making Test part A; TMT-B: Trail Making Test part B; TUG: Timed Up and Go Test; WCST: Wisconsin Card Sorting Test.

**Table 5:** Correlation between cognitive function test, olfactory identification test, balance capability tests, and muscle strength tests in the control group.

	MoCA	TMT-A	TMT-B	WCST (CA)	WCST (PEN)	QIDS	SAS
Open essence	−0.075	−0.133	0.067	0.093	−0.083	−0.111	0.106
OLST (eyes open)	0.092	−0.217	−0.114	0.069	−0.008	−0.114	**−0.229∗**
OLST (eyes closed)	−0.026	−0.050	0.151	−0.013	0.007	−0.197	−0.057
TUG	0.056	0.109	−0.111	−0.006	0.003	0.095	0.062
IPS	0.159	**−0.250∗**	−0.146	0.086	−0.121	−0.142	−0.205
Grip strength	−0.146	−0.176	**−0.219∗**	0.121	−0.092	−0.117	**−0.238∗**
Knee extension strength	−0.038	−0.204	**−0.258∗**	0.180	**−0.256∗**	−0.090	−0.216
Knee extension endurance	−0.039	−0.154	**−0.223∗**	0.209	**−0.272∗**	−0.033	**−0.240∗**

^∗^ *P* < 0.05, ^∗∗^*P* < 0.01, and ^∗∗∗^*P* < 0.001. CA: categories achieved; IPS: index of postural stability; MoCA: Montreal Cognitive Assessment; OLST: one-leg standing time; PEN: perseverative errors of Nelson; QIDS: Quick Inventory of Depressive Symptomatology Self-Report; SAS: Starkstein Apathy Scale; TMT-A: Trail Making Test part A; TMT-B: Trail Making Test part B; TUG: Timed Up and Go Test; WCST: Wisconsin Card Sorting Test.

**(a):** 

	MoCA∗		TMT-A∗∗	
	β	*P*	β	*P*
Age	−0.061	0.466	0.034	0.700
Male	−0.102	0.301	−0.043	0.675
Education >12 y	−0.043	0.590	−0.035	0.679
Diabetes	−0.196	0.083	−0.014	0.907
Hypertension	−0.019	0.809	0.149	0.070
ApoE ε4 carrier	0.040	0.584	0.033	0.668
Body mass index	0.006	0.949	−0.082	0.415
Open essence	0.142	0.062	**−0.161**	**0.043**
IPS	**0.329**	**<0.001**	**−0.260**	**0.006**
Knee extension strength	−0.036	0.758	−0.202	0.103

**(b):** 

	TMT-B∗∗		WCST (CA)	
	β	*P*	β	*P*
Age	0.092	0.268	−0.125	0.170
Male	0.089	0.367	0.144	0.180
Education >12 y	0.093	0.244	0.044	0.617
Diabetes	0.099	0.376	−0.107	0.379
Hypertension	0.070	0.372	0.100	0.241
ApoE ε4 carrier	0.022	0.762	0.042	0.596
Body mass index	−0.114	0.238	0.092	0.377
Open essence	−0.092	0.225	0.056	0.495
IPS	**−0.324**	**<0.001**	**0.319**	**0.001**
Knee extension strength	**−0.239**	**0.044**	0.012	0.926

**(c):** 

	WCST (PEN)∗∗		QIDS∗∗	
	β	*P*	β	*P*
Age	0.105	0.229	**−0.190**	**0.031**
Male	−0.141	0.169	−0.158	0.126
Education >12 y	−0.044	0.599	−0.033	0.696
Diabetes	0.215	0.066	**0.290**	**0.014**
Hypertension	−0.058	0.480	0.058	0.479
ApoE ε4 carrier	−0.001	0.987	**0.194**	**0.012**
Body mass index	−0.094	0.348	0.038	0.703
Open essence	−0.080	0.307	−0.155	0.052
IPS	**−0.253**	**0.007**	−0.141	0.134
Knee extension strength	−0.097	0.429	0.030	0.811

**(d):** 

	SAS	
	β	*P*
Age	**−0.302**	**0.001**
Male	−0.015	0.886
Education >12 y	−0.103	0.224
Diabetes	0.173	0.145
Hypertension	0.019	0.817
ApoE ε4 carrier	−0.005	0.952
Body mass index	−0.108	0.288
Open essence	−0.020	0.806
IPS	−0.093	0.323
Knee extension strength	**−0.309**	**0.014**

^∗^Cubed variables and ^∗∗^log-transformed variables are used for the analyses. ApoE: apolipoprotein E; CA: categories achieved; IPS: index of postural stability; MoCA: Montreal Cognitive Assessment; PEN: perseverative errors of Nelson; QIDS: Quick Inventory of Depressive Symptomatology Self-Report; SAS: Starkstein Apathy Scale; TMT-A: Trail Making Test part A; TMT-B: Trail Making Test part B; WCST: Wisconsin Card Sorting Test.

**(a):** 

	MoCA∗			
	Controls		Type 2 diabetes	
	β	*P*	β	*P*
Age	0.055	0.648	−0.170	0.145
Male	−0.152	0.309	−0.065	0.667
Education >12 y	−0.045	0.702	−0.014	0.898
ApoE ε4 carrier	0.017	0.882	0.105	0.331
Open essence	−0.113	0.335	**0.364**	**0.001**
IPS	0.230	0.051	**0.428**	**<0.001**
Knee extension strength	0.034	0.824	−0.143	0.361

	TMT-A∗∗			
	Controls		Type 2 diabetes	
	β	*P*	β	*P*
Age	−0.030	0.792	0.223	0.083
Male	−0.147	0.302	0.086	0.603
Education period	0.023	0.840	−0.105	0.391
ApoE ε4 carrier	0.090	0.405	−0.079	0.503
Open essence	−0.157	0.160	−0.141	0.234
IPS	**−0.231**	**0.039**	**−0.254**	**0.049**
Knee extension strength	−0.213	0.146	−0.141	0.410

	TMT-B∗∗			
	Controls		Type 2 diabetes	
	β	*P*	β	*P*
Age	0.069	0.567	0.179	0.105
Male	−0.055	0.714	0.199	0.167
Education period	0.085	0.471	0.124	0.243
ApoE ε4 carrier	−0.019	0.865	0.032	0.756
Open essence	0.067	0.561	**−0.264**	**0.012**
IPS	−0.125	0.281	**−0.434**	**<0.001**
Knee extension strength	−0.205	0.180	−0.147	0.321

**(b):** 

	WCST (CA)			
	Controls		Type 2 diabetes	
	β	*P*	β	*P*
Age	−0.077	0.526	−0.220	0.086
Male	0.178	0.240	0.190	0.252
Education >12 y	0.020	0.865	0.030	0.806
ApoE ε4 carrier	0.031	0.784	0.077	0.516
Open essence	0.082	0.485	0.058	0.625
IPS	0.116	0.323	**0.379**	**0.004**
Knee extension strength	0.027	0.863	−0.121	0.481

	WCST (PEN)∗∗			
	Controls		Type 2 diabetes	
	β	*P*	β	*P*
Age	0.211	0.072	0.060	0.645
Male	−0.189	0.193	−0.174	0.309
Education >12 y	0.034	0.768	−0.048	0.702
ApoE ε4 carrier	0.005	0.961	0.005	0.967
Open essence	−0.106	0.348	−0.084	0.493
IPS	−0.107	0.341	**−0.334**	**0.013**
Knee extension strength	−0.079	0.593	<0.001	0.999

	QIDS∗∗			
	Controls		Type 2 diabetes	
	β	*P*	β	*P*
Age	−0.167	0.159	**−0.302**	**0.024**
Male	−0.121	0.407	−0.126	0.462
Education >12 y	−0.123	0.285	0.057	0.649
ApoE ε4 carrier	**0.232**	**0.040**	0.147	0.233
Open essence	−0.108	0.342	−0.222	0.073
IPS	−0.144	0.209	−0.178	0.177
Knee extension strength	−0.057	0.705	0.062	0.727

**(c):** 

	SAS			
	Controls		Type 2 diabetes	
	β	*P*	β	*P*
Age	**−0.246**	**0.037**	**−0.299**	**0.024**
Male	−0.169	0.243	0.137	0.419
Education >12 y	−0.025	0.828	−0.112	0.371
ApoE ε4 carrier	−0.018	0.869	−0.002	0.989
Open Essence	0.081	0.471	−0.075	0.538
IPS	−0.194	0.086	<0.001	0.999
Knee extension strength	−0.166	0.264	**−0.375**	**0.035**

^∗^Cubed variables and ^∗∗^log-transformed variables are used for the analyses. ApoE: apolipoprotein E; CA: categories achieved; IPS: index of postural stability; MoCA: Montreal Cognitive Assessment; PEN: perseverative errors of Nelson; QIDS: Quick Inventory of Depressive Symptomatology Self-Report; SAS: Starkstein Apathy Scale; TMT-A: Trail Making Test part A; TMT-B: Trail Making Test part B; WCST: Wisconsin Card Sorting Test.

**Table 8:** Comparison of cognitive function tests, odor identification tests, and physical function tests in age-, sex-, and years of education-matched cohort.

	Controls	Type 2 diabetes	*P*
	(n = 44)	(n = 44)	
Age (y)	53 ± 6	53 ± 7	0.949
Female, n (%)	25 (57)	25 (57)	1.000
Education (y)	14 (12 – 16)	14 (12 – 16)	0.313
ApoE *ε*4 allele carrier, n (%)	13 (30)	4 (9)	0.014
Cognitive function			
MoCA (points)	27 (26 – 28)	25 (22 – 26)	< 0.001
TMT-A (s)	25.0 (21.9 – 29.8)	32.1 (26.5 – 38.4)	0.001
TMT-B (s)	60.0 (49.2 – 77.8)	85.6 (60.6 – 108.5)	< 0.001
WCST			
CA (points)	4 (3 – 5)	2 (1 – 5)	0.028
PEN (points)	4 (2 – 7)	10 (4 – 16)	< 0.001
QIDS (points)	3 (2 – 6)	5 (4 – 8)	< 0.001
SAS (points)	9 ± 5	13 ± 6	< 0.001
Olfactory function			
Open Essence (points)	10 (9 – 11)	9 (8 – 10)	0.010
Physical function			
OLST (eyes open) (sec)	120 (120 – 120)	120 (40 – 120)	< 0.001
OLST (eyes closed) (s)	20 (9 – 29)	7 (4 – 16)	< 0.001
TUG (s)	5.0 ± 0.7	6.1 ± 0.7	< 0.001
IPS	1.94 (1.83 – 2.02)	1.64 (1.43 – 1.78)	< 0.001
Grip strength (kg)	31 (26 – 41)	26 (21 – 40)	0.046
Knee extension strength (Nm/kg)	214 ± 43	164 ± 48	< 0.001
Knee extension endurance (J)	1081 (899 – 1416)	890 (707 – 1179)	0.006

Data are mean ± SD or median (interquartile range). CA: categories achieved; IPS: index of postural stability; MoCA: Montreal Cognitive Assessment; OLST: one-leg standing time; PEN: perseverative errors of Nelson; QIDS: Quick Inventory of Depressive Symptomatology Self-Report; SAS: Starkstein Apathy Scale; TMT-A: Trail Making Test part A; TMT-B: Trail Making Test part B; TUG: Timed Up and Go Test; WCST: Wisconsin Card Sorting Test.

## Data Availability

The data used to support the findings of this study are available from the corresponding author upon request.
